# Using seasonal landscape models to predict space use and migratory patterns of an arctic ungulate

**DOI:** 10.1186/s40462-019-0162-8

**Published:** 2019-06-06

**Authors:** A. P. Baltensperger, K. Joly

**Affiliations:** 4175 Geist Rd, National Park Service, Fairbanks, AK 99709 USA

**Keywords:** Alaska, Caribou, Climate, Ecological niche model, Infrastructure, Machine learning, Potential evapotranspiration, *Rangifer tarandus*, Snow, Stochastic gradient boosting

## Abstract

**Background:**

Caribou in the Western Arctic Herd undertake one of the longest, remaining intact migrations of terrestrial mammals in the world. They are also the most important subsistence resource for many northern rural residents, who rely on the caribou’s migratory movements to bring them near for harvest. Migratory geography has never been static, but subsistence harvesters have reported recent shifts in migration away from areas where they traditionally occurred. The reasons behind these changes are not well-understood, but may be related to rapid climate change and anthropogenic disturbances.

**Methods:**

To predict changes in distribution and shifting migratory areas over the past decade, we used GPS telemetry data from adult females to develop predictive ecological niche models of caribou across northwestern Alaska. We employed the machine-learning algorithm, TreeNet, to analyze interactive, multivariate relationships between telemetry locations and 37 spatial environmental layers and to predict the distributions of caribou during spring, calving season, insect-harassment season, late summer, fall, and winter from 2009 to 2017. Model results were analyzed to identify regions of repeated predicted use, quantify mean longitude, predict land cover selection, and track migratory changes over time.

**Results:**

Distribution models accurately predicted caribou at a spatially-explicit, 500-m scale. Model analyses identified migratory areas that shifted annually across the region, but which predicted 4 main areas of repeated use. Niche models were defined largely by non-linear relationships with coastally-influenced, climatic variables, especially snow-free date, potential evapo-transpiration, growing season length, proximity to sea ice, winter precipitation and fall temperature. Proximity to roads and communities were also important and we predicted caribou to generally occur more than 20–100 km from these features.

**Conclusions:**

Western Arctic Herd caribou were predicted to occur in warmer, snow-free and treeless areas that may provide conditions conducive for efficient travel and foraging. Rapidly changing seasonal climates and coastal influences that determine forage availability, and human impediments that slow or divert movements are related to geographically and phenologically dynamic migration patterns that may periodically shift caribou away from traditional harvest areas. An enhanced understanding of the geographic behavior of caribou over time could inform traditional harvests and help conserve important Western Arctic caribou migratory areas.

**Electronic supplementary material:**

The online version of this article (10.1186/s40462-019-0162-8) contains supplementary material, which is available to authorized users.

## Background

Animals undertake a variety of movements based on both intrinsic and external factors including climate, food availability, competition, and predation risk [1, 2]. Movement decisions are made at a range of scales from individual steps (length and direction) [3, 4] to the selection of patches [5, 6], and seasonal migratory route finding through regional landscapes [7]. Long-distance migrations have been identified in a variety of North American ungulates including pronghorn (*Antilocapra americana* [8], elk (*Cervus canadensis* [9]*,* mule deer (*Odocoileus hemionus*) [5, 10, 11], bison (*Bison bison*) [12], moose (*Alces alces*) [13] and caribou (*Rangifer tarandus*) [14]. Migration is often defined as the cyclical movement of populations between disjunct ranges as a means of increasing fitness as populations seek quality forage and avoid seasonally abundant pests and predators [15, 16].

The prevalence and length of long-distance terrestrial migrations have been steadily declining and phenology has been altered due to a range of limiting factors. Impediments to movement include barriers such as transportation corridors [16–19], fences [8], urban and industrial development [20, 21], unavailable forage and habitat [5], and unstable ice and snow conditions [22–24]. These obstacles cause animals to expend additional time and energy to navigate. Disturbances to seasonal movements of herds may also include wildfires, high densities of hunters, predators, or development near migratory bottlenecks or stopover sites [25–28]. Because migration routes are difficult to restore once interrupted, identifying and conserving important migratory areas now should be a top priority for land managers [7, 18, 25].

**Fig. 1:**
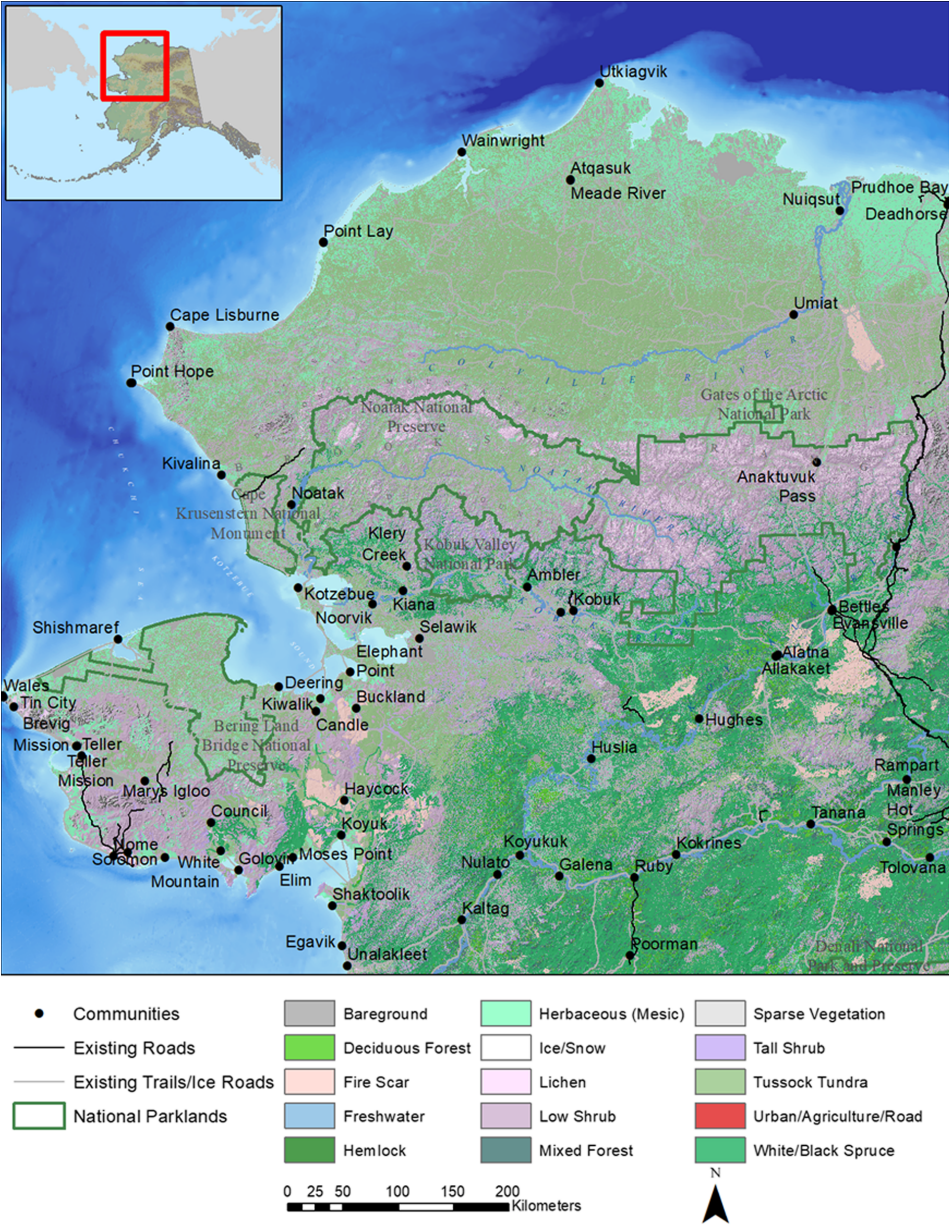
Study area map. Map of northwestern Alaska including its placement relative to the state of Alaska (inset), geographic landmarks, anthropogenic features, National Park Service boundaries (green), and land cover ecotypes. The extent of the map indicates the study area

Alaska includes over 30 herds of caribou distributed throughout the state, although many of these are smaller herds that undertake only local migrations or none at all [29]. Four large migratory herds range across the Brooks Range in northern Alaska. The largest among these is the Western Arctic Herd (WAH), comprising 259,000 individuals in 2017 and distributed seasonally across over 360,000 km^2^ of northwestern Alaska (Fig. 1) [30, 31]. After reaching its historic nadir of 75,000 individuals in 1976, the WAH grew rapidly to nearly 490,000 animals, making it one of the largest caribou herds in the world in 2003 [32]. By 2016, the herd had declined 60% to 201,000 animals [30]. During their typical annual migratory cycle, WAH caribou travel from wintering grounds on and near the Seward Peninsula, over the Brooks Range to calve on the North Slope. They then make their way towards the coast to escape insect harassment in mid-summer before returning south again in fall to their wintering grounds. The straight-line, round-trip distance between their winter and calving areas exceeds 1000 km, which is among the longest terrestrial migrations on the planet [25]. Caribou wander extensively and their total annual movements exceed 4400 km [33].

The WAH is one of the most important subsistence resources in northwest Alaska, providing local rural residents from over 40 communities with 11,000 caribou annually [27, 32]. During the 2000s, rural residents began to observe shifts in migrations away from communities, changes in herd phenology, and a decrease in the number and quality of caribou available for harvest [34]. On average, sport hunters comprised roughly 5% of the total harvest each year until 2016 [27, 32, 35]. These user groups have come into conflict as traditional harvesters have perceived aircraft noise, often associated with sport hunting to be disruptive to caribou movements [36, 37]. However, research has not found influence of hunter activity on herd behavior at caribou movements at broad scales in the Noatak River Valley [27]. In an effort to stem the herd’s decline, managers instituted closures for sport hunting and limits on traditional harvest in 2016 [33]. At this time, the herd is showing signs of recovering survival and recruitment rates [30]. The health of the herd and the location of migratory movements from year to year will determine the success of subsistence harvesters who rely on caribou for their own food security.

However, human development has been shown to influence caribou distributions during all seasons [16, 20]. Transportation and industrial corridors such as frequently-traveled roads and railways as well as pipelines and seismic lines can act as barriers by delaying or interrupting crossing, especially when perpendicular to the direct of travel [16, 38]. Roads and railways are also direct sources of mortality through collisions [17, 21] and increased vigilance around these obstacles may also reduce foraging efficiency, harming individual health. Wilson et al. documented a delay in collared, female WAH caribou by an average of 33 days while crossing the Red Dog Mine road [16]. The distribution of the CAH during calving in the Prudhoe Bay area has also been shown to be inversely related to road density and to shift at least 5 km away from pipelines and roads with even limited traffic levels of 100–200 vehicles/day, although they may use these areas during the insect relief season [20, 39]. Other disturbances associated with concentrations of humans that may affect caribou include noise disturbance from water- and aircraft [37], and industrial machinery [40].

In addition to anthropogenic impediments, caribou distribution patterns in northern Alaska are related to a collection of climate-induced effects including increased fire frequency and vegetative biomass, which act to convert lichen tundra to shrub-lands [41], reducing available winter forage [22, 28]. Global climate change is already resulting in stronger and more intense storms and deeper snow-packs [42], but also more frequent rain-on-snow events that increase snow-pack density [43, 44]. Such conditions make it difficult for caribou to access lichens and to travel efficiently in winter and spring [45]. The size of the WAH has also been shown to vary with changes in the PDO (Pacific Decadal Oscillation) [45], where positive phases (warmer conditions) tend to correlate with herd growth and larger population sizes versus smaller, declining populations during negative phases (colder conditions) [45]. Furthermore, the dramatic decrease in sea ice extent observed over the past decades has also been hypothesized to affect caribou through changes in weather patterns, especially during the insect-avoidance season [22, 46]. The combined effects of shifting mean temperatures and precipitation, prevailing winds, insect phenology, and plant growth, affect the forage availability, predation rates, calf recruitment, individual body condition and ultimately the population distribution of the WAH throughout the year [45].

Quantifying seasonal ranges, defining migratory habitat and detecting changes in caribou distributions and migration geography over time are among the stated monitoring objectives for the National Park Service [33], and providing up-to-date information to local communities may help them to adapt to a rapidly changing Arctic [34]. Despite regular research to estimate herd size, studies to quantify and track migratory areas in northern Alaska have only been conducted for the Central Arctic Herd (CAH) [7]. The factors behind WAH population dynamics, migratory patterns, and overall health are numerous and interactive, so deciphering these drivers and identifying impediments requires a multivariate approach that can incorporate dozens of variables. Here we use the machine learning algorithm, TreeNet, to analyze GPS telemetry data for the WAH over 8 years. This approach uses associations between caribou and the environment to develop accurate predictive models, identify important system predictors, and define non-linear responses. Our goals were to 1) model caribou seasonal distributions and change, 2) relate caribou occurrence to climatic, anthropogenic, and topographic predictors, and 3) to identify migratory areas predicted to have been used consistently by the WAH over the past decade. We hope these results may help to conserve an important regional subsistence resource and promote the preservation of one of the largest and longest remaining terrestrial migrations in the world.

## Methods

### Data collection

Over the past decade, 211 adult female caribou belonging to the WAH were captured in Kobuk Valley National Park, Alaska and fitted with GPS telemetry collars (Telonics TGW-4680, Mesa, Arizona, USA) following protocols approved by a State of Alaska Institutional Animal Care and Use Committee (IACUC 0040–2017-40) [33]. Only adult (> 2 years old) female caribou were fitted with collars, which collected position data every 8 h throughout the year (1095 relocations per caribou per year).

### Model development

We used the machine learning algorithm, TreeNet (Salford Systems, Inc., San Diego, CA), a type of stochastic gradient boosting, to analyze associations between locational records of caribou and dozens of climatic, topographic, and anthropogenic predictors (Table 1), and to spatially model herd distribution during biologically significant seasons over 8 years. This approach, which uses iterative tree-building to reduce overall error and collinearity among points and predictors, is known as “boosting” and differs from traditional frequentist statistical analyses in its analysis and assumptions. Boosted decision tree analyses are capable of quantifying complex, non-linear relationships among many interacting categorical and continuous predictors, and are insensitive to outliers and missing data [47, 48]. Because TreeNet is a data-driven approach and not specifically designed to test a priori hypotheses, datasets need not conform to assumptions of normality and independence, and so do not require prior data manipulation. We chose this approach over parametric analyses like dynamic Brownian Bridge and step-selection analyses, that are constrained by a priori assumptions, for its ability to incorporate numerous, interacting, non-linear, environmental relationships in predicting the distribution of species across broad landscapes. While this approach is unable to determine causation between predictors and the response, it is adept at producing accurate predictions and useful for exploring the relative importance and non-linear responses of predictors.

**Table 1:** Predictors and variable importance scores in seasonal distribution models of the Western Arctic Herd

Included 2010 Predictors (units)	Source	Spring	Calving	Insect Relief	Late Summer	Fall	Winter
Active Layer Thickness*∆ (m)	www.snap.uaf.edu/tools/data-downloads	31.0	15.1	16.6	15.6	31.4	18.4
Aspect (°)	ned.usgs.gov	27.6	13.5	11.3	15.2	24.6	10.6
Coast Distance (m)	www.asgdc.state.ak.us	43.3	23.6	18.3	24.0	42.9	34.8
Elevation (m)	ned.usgs.gov	30.6	13.2	14.3	15.7	32.3	16.1
Freeze Date*∆ (Julian date)	www.snap.uaf.edu/tools/data-downloads	9.7	4.2	3.1	5.3	17.9	7.3
Thaw Date*∆ (Julian date)	www.snap.uaf.edu/tools/data-downloads	17.2	5.5	5.1	4.1	12.3	8.5
Fire History∆ (year)	agdc.usgs.gov/data/projects/fhm	14.6	3.8	2.5	3.2	14.1	15.9
Lake Distance (m)	nhd.usgs.gov	31.5	15.3	11.6	17.6	29.4	15.5
Growing Season Length*∆ (days)	www.snap.uaf.edu/tools/data-downloads	32.8	9.6	6.2	10.0	51.2	16.0
Ground Temperature*∆ (°C)	www.snap.uaf.edu/tools/data-downloads	36.6	14.8	11.7	15.0	64.6	21.3
NDVI Greenup Rate∆ (−0.2˗˗1.0)	nhd.usgs.gov	25.6	13.7	11.7	16.1	24.8	10.7
Max NDVI∆ (−0.2˗˗1.0)	alaska.portal.gina.alaska.edu/search	22.2	5.4	7.9	17.9	27.5	15.6
Potential Evapotranspiration*∆ (mm/yr)	arcticlcc.org/products/spatial-data	69.0	100.0	100.0	28.1	100.0	35.5
Permafrost Probability (%)	[91]	18.9	10.6	10.2	16.0	32.9	18.1
Winter Precipitation*∆ (mm)	www.snap.uaf.edu/tools/data-downloads	100.0	13.0	8.0	57.6	47.1	25.3
Summer Precipitation*∆ (mm)	www.snap.uaf.edu/tools/data-downloads	26.7	12.7	14.2	21.4	34.0	26.9
Spring Precipitation*∆ (mm)	www.snap.uaf.edu/tools/data-downloads	31.5	25.4	6.7	15.8	27.8	19.1
Fall Precipitation*∆ (mm)	www.snap.uaf.edu/tools/data-downloads	35.7	12.5	35.5	20.1	44.4	21.1
Stream Distance (m)	nhd.usgs.gov	30.1	13.9	10.8	15.8	26.8	13.9
Road Distance (m)	www.asgdc.state.ak.us	60.9	31.1	26.5	27.9	44.3	39.1
Max (Mar) Sea Ice Distance*∆ (m)	[92]	53.4	18.7	12.9	17.1	41.8	30.9
Min (Sep.) Sea Ice Distance*∆ (m)	[92]	58.3	47.5	16.4	21.9	43.2	37.4
Slope (⎕)	ned.usgs.gov	28.4	13.3	12.2	17.9	34.8	13.0
Winter Snow Day Fraction*∆ (%)	www.snap.uaf.edu/tools/data-downloads	11.6	2.8	14.1	24.7	20.6	100.0
Summer Snow Day Fraction*∆ (%)	www.snap.uaf.edu/tools/data-downloads	18.1	10.0	6.1	9.3	14.8	10.0
Spring Snow Day Fraction*∆ (%)	www.snap.uaf.edu/tools/data-downloads	25.2	7.2	7.0	9.0	16.1	13.6
Fall Snow Day Fraction*∆ (%)	www.snap.uaf.edu/tools/data-downloads	26.6	7.0	6.9	100.0	23.7	19.9
Snow-free Date*∆ (Julian date)	[62]	64.2	10.4	9.3	11.7	27.7	25.8
Winter Temperature*∆ (°C)	www.snap.uaf.edu/tools/data-downloads	25.8	17.4	9.0	15.6	29.5	28.1
Summer Temperature*∆ (°C)	www.snap.uaf.edu/tools/data-downloads	28.3	15.4	7.6	10.2	28.5	12.0
Spring Temperature*∆ (°C)	www.snap.uaf.edu/tools/data-downloads	22.0	12.1	9.8	10.5	24.7	12.6
Fall Temperature*∆ (°C)	www.snap.uaf.edu/tools/data-downloads	65.4	20.8	9.3	13.7	26.6	21.4
Distance to Trails (m)	www.asgdc.state.ak.us	35.7	16.1	17.0	18.9	34.2	23.6
Terrain Ruggedness 17 × 17 (0–1)	ned.usgs.gov	28.2	20.6	18.7	21.4	28.8	33.7
Terrain Ruggedness 3 × 3 (0–1)	ned.usgs.gov	26.5	13.8	12.1	16.1	24.4	10.5
Community Distance (m)	www.asgdc.state.ak.us	43.4	14.9	11.9	15.1	43.6	13.6
Wetland Distance (m)	https://www.fws.gov/wetlands/Data/Data-Download.html	25.4	13.7	11.9	15.6	30.7	13.8

Predictors denoted with an * are decadal means, and ∆ indicates dynamic predictors that change annually. Active layer thickness is the depth of ground that thaws and freezes each year. Freeze and thaw dates refer to the mean annual date of first freeze (or thaw) during 2010–2020. Fire history denotes the year of the most recent fire in a given area. Growing season length is defined as the number of days between the date of first thaw and date of first freeze. Ground temperature refers to the modeled mean annual ground temperature. NDVI (Normalized Difference Vegetation Index) is an infrared-based measure of landscape greenness indicative of biomass; green-up rate and maximum NDVI are annual metrics calculated from NDVI. Potential evapotranspiration (PET) here is the amount of evaporation that would occur over a year if a sufficient water source were available. Seasonal climate variables are decadal averages of downscaled seasonal totals. Seasons are grouped as follows: Spring (Mar.-May), Summer (Jun.-Aug.), Fall (Sep.-Nov.), Winter (Dec.-Feb.). Permafrost probability describes the distribution of near-surface permafrost. Distance refers to the shortest Euclidean distance to the nearest feature. Snow day fraction is an average of the percentage of days with snow on the ground. Terrain ruggedness predictors were calculated from slope and elevation using 2 moving window scales of 3 and 17 m, respectively. Predictor importance values are out of 100.0 with the top predictor always receiving the maximum

We developed landscape distribution models for northwestern Alaska based on GPS locations attributed with 37 environmental predictors for 6 seasons (averaged over the course of 8 years) as well as each year for the spring and fall migratory seasons (Table 1). For consistency, we defined seasons based on previously determined date ranges (Joly and Cameron 2017). We developed distribution models for 6 biologically-relevant seasons: spring (April 1–May 27), calving (May 28–June 14), insect-relief (June-15-July 14), late summer (July 15–August 31), fall (September 1–November 30), and winter (December 1–March 31). Training datasets for each season consisted of the recorded locations for all individuals across all years for which we had data. Because models are based on presence-only datasets, we used sets of randomly-distributed pseudo-absences in lieu of observed absences, which are not inherent to telemetry datasets. We determined the numbers of pseudo-absences in each model by multiplying the number of presences for each season or year by the study area extent and dividing by the minimum convex hull area of the presence locations. We did this to ensure that the relative densities of presences and pseudo-absences were consistent across seasons and years to avoid ‘swamping’ the presence set. We defined the study area as the portion of northwest Alaska encompassing the full extent of telemetry locations from 2010 to 2017 (e.g., Fig. 1).

Together presences and pseudo-absences composed the training dataset for each model. We attributed these points with the 37 environmental predictors using the ‘extract’ function in R 3.5.1 (R Development Core Team 2018). Environmental predictors in the models included climatic, anthropogenic, geographic and topographic predictors known or hypothesized to affect caribou ecology in some way (Table 1). We then analyzed the training data, using TreeNet, to iteratively classify non-linear relationships between predictors and the response (Relative Index of Occurrence; RIO). We used model classifications to make predictions regarding the spatio-temporal distributions of caribou, to identify important predictors in the models, and to quantify thresholds of influence in the non-linear contribution of those predictors to the response. TreeNet functions by first developing a single decision tree to estimate the main effect on the response by accounting for the largest proportion of variance in a system [47, 49]. In subsequent, iterative steps, additional trees are ‘grown’ to explain the residual error that remains from previous trees [48, 49]. TreeNet builds thousands of trees, uses recursive data sampling, and predictors enter the model in a gradual, non-linear fashion, with different splitting rules each time they are used. This iterative resampling helps to minimize the effect of issues such as model over-fitting [47, 48] and spatial autocorrelation [50, 51]. Stochastic gradient boosting algorithms also automatically include interactions among all predictors in a model [48, 49, 52, 53]. Boosting improves model accuracy by averaging contribution from numerous satisfactory but imperfect models in a successive fashion rather than attempting to find a single parsimonious model to approximate the system [48, 49, 53, 54].

We varied the number of trees (1000–2000), number of nodes per tree (6–20), and minimum cases per terminal node (2–50) in TreeNet to identify the best model with the highest area under the receiver operators curve (AUC) and the lowest misclassification rate. We excluded the poorest performing predictors in successive modeling attempts to improve predictive accuracy, but the full predictor set proved to be most accurate. We used the ‘balanced’ option to ensure equal contribution to the model from unequal class sizes in the response variable. We validated models using two internal means, using the out-of-bag data withheld during the tree building process, and also by withholding 20% of the training data (test subset) before model construction. Models were graded as “excellent” (1.0 > % Correct > 0.90), “good” (0.90 > % Correct > 0.80), “fair” (0.80 > % Correct > 0.70). We also used telemetry data from 2018 as an independent dataset to validate the predictive spatial accuracy of models by calculating the mean and standard deviation of residuals (1 – RIO).

For each model, TreeNet developed a unique algorithm to describe patterns in the data, which was then ‘scored’ to a regularly-spaced grid of points (500-m resolution) across the study area that was also attributed with the same environmental variables. Predictions at points were smoothed using the inverse-distance weighting function to produce a continuous raster depicting the RIO for caribou in northwestern Alaska. This process was repeated to produce distribution models for each of the 6 caribou seasons pooled across years and 8 individual fall and spring models for each year between 2010 and 2017. TreeNet also produced partial dependence plots for each predictor. These are model-based simulations that chart the non-linear relationship of the response over the range of each predictor variable while controlling for the other variables [55]. Partial dependence plots are useful for depicting trends and identifying threshold levels in predictors which correspond with the predicted presence or absence of caribou. We used partial dependence plots to identify thresholds and delineate data ranges in environmental predictors that were associated with the predicted occurrence of caribou. Each of these ranges represents a single plane of realized niche space composing a theoretical n-dimensional hypervolume of available environmental conditions that constrain the potential niche of caribou [56].

### Analyses

To predict the most common migratory areas, we converted the annual distribution models for the fall and spring seasons to binary models using the ‘reclassify’ tool in ArcGIS 10.5. Predicted presences were differentiated from predicted absences using the threshold between 0 and 1 that minimized the total error rate for each model [57]. We calculated the total area of predicted presence for each year by multiplying the number of presence pixels by the pixel area (500 m^2^). We also calculated the mean latitude and mean longitude of observed telemetry locations in spring and fall to quantify geographic shifts among years. For both migratory seasons, we summed models across years to determine the number of years each pixel was predicted to have been used by caribou. From these, we identified primary migratory areas that were predicted to have been used repeatedly by caribou across the study area in both spring and fall. We also used ArcGIS to calculate the mean pixel values of the top environmental predictors in areas of predicted presence and absence in spring and fall. We intentionally excluded a spatial land cover predictor from the model to subsequently evaluate proportional land cover use independently in relation to predicted distribution areas. We related predicted presence areas during migratory seasons with a comprehensive land cover map of the region [58] using the tabulate area tool in ArcGIS. We calculated landcover selection by dividing the percentage of pixels predicted as presences in each cover type by the percentage of pixels of that cover type available in the study area.

## Results

### Pooled seasonal models

**Fig. 2:**
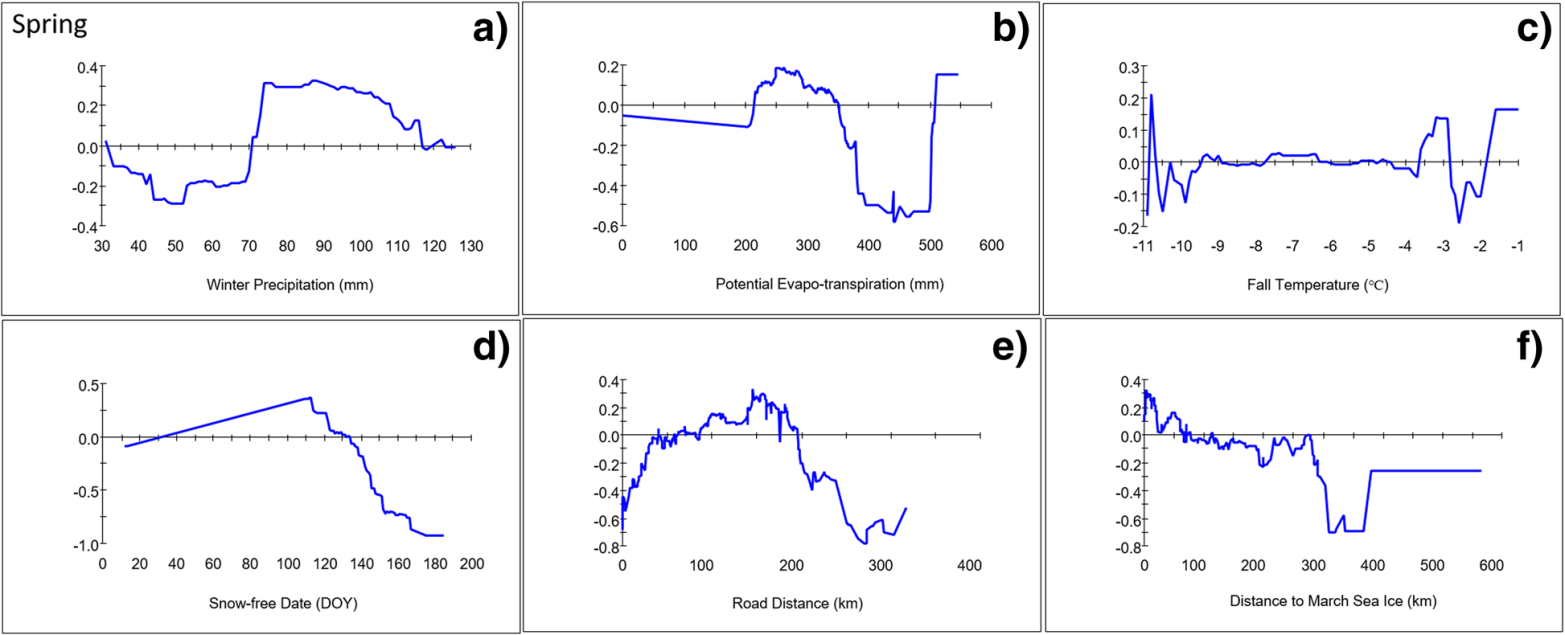
Partial-dependence plots for the top 6 predictors, mean winter precipitation (**a**), potential evapotranspiration (**b**), mean fall temperature (**c**), mean snow-free date (**d**), distance to nearest road (**e**), and distance to mean maximum sea ice extent (**f**), for the pooled (2010–2017) spring caribou distribution model

**Fig. 3:**
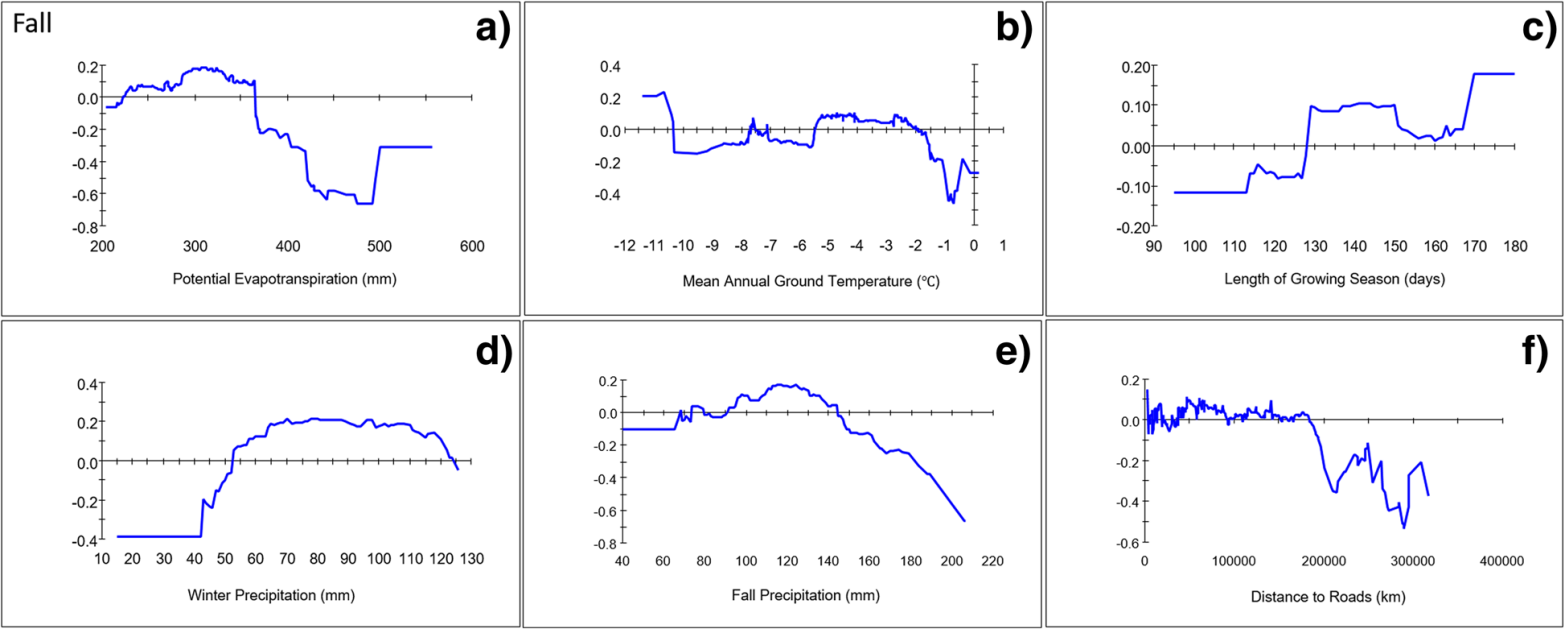
Partial-dependence plots for the top 6 predictors, potential evapotranspiration (**a**), mean annual ground temperature (**b**), mean length of growing season (**c**), mean winter precipitation (**d**), mean fall precipitation (**e**), and distance to nearest road (**f**), for the pooled (2010–2017) fall caribou distribution model

**Fig. 4:**
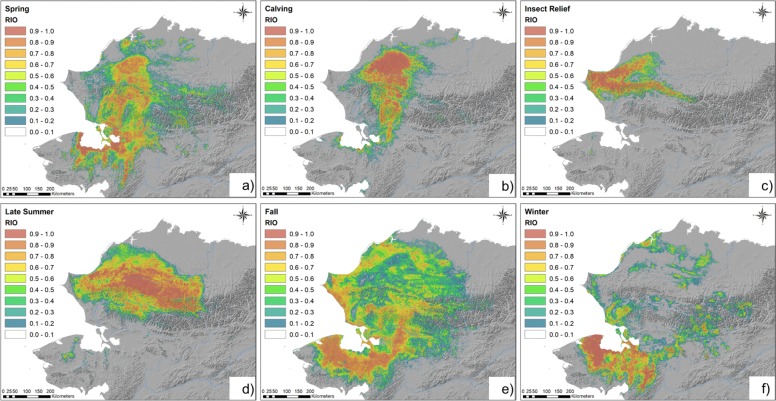
Modeled seasonal distributions of Western Arctic Herd caribou. Models were developed for the spring (**a**), calving (**b**), insect-relief (**c**), late-summer (**d**), fall (**e**), winter (**f**) seasons during 2010–2017. Warm colors indicate areas of high predicted Relative Index of Occurrence (RIO), cool colors indicate areas of low predicted RIO

GPS collars recorded locations of WAH caribou from September 7, 2010 to November 30, 2017 resulting in a total of 465,807 occurrence records. Seasonal models, pooled across years, predicted regions of use by the WAH during the spring, calving, insect-relief, late summer, fall, and winter seasons. Based on AUC scores and overall percent correct, model accuracies ranged from ‘good’ to ‘excellent’ in their predictions of the spatio-temporal distribution of caribou in the WAH. Environmental predictors were ranked to show the most influential predictors in the model (Table 1) and partial dependence plots Figures 2, 3, Additional file 1) indicated ranges of each predictor that were associated and disassociated with the predicted distribution of caribou in the models (Fig. 4).

The spring model predicted the presence of caribou in a belt of land around the coast of Kotzebue Sound and a band extending northward across the Kobuk River between the communities of Noorvik and Kobuk (Fig. 4a). Three swaths of predicted presence diffused north over the Baird Mountains and across the breadth of Noatak National Preserve (NOAT). Areas of high RIO extended over the Brooks Range across numerous mountain passes before converging on the North Slope. The overall accuracy for the best pooled spring model was deemed ‘good’ (AUC = 0.95; Overall Accuracy_test_ = 88.45%; Mean Residual_2018_ = 0.39 ± 0.30) at a presence/absence threshold of 0.61. Winter precipitation was the most important predictor in the spring distribution model (Table 1), which predicted caribou to occur in areas with more than 70 mm of winter precipitation (Fig. 2a). The mean precipitation in areas predicted as presences was 84.8 mm versus 66.8 mm in absence areas. PET (potential evapo-transpiration) was also highly predictive of the presence of caribou (Table 1), especially in areas where the mean evaporative potential was 220–350 mm, but the 2 were negatively correlated when PET exceeded 350 mm (Fig. 2b). The mean evaporative potential in predicted presence areas was 293.0 mm versus 360.0 mm across absence areas. Models also generally predicted caribou to occur in areas where mean decadal fall temperatures were above − 4 °C (Fig. 2c) and where average snow melt occurred between April 20 and May 10 (Fig. 2d). Local snow melt timing varied by as much as 2 months across ridgelines in areas frequented by caribou. The model showed a tendency for caribou to be negatively associated with roads at distances within 40 km (mean = 89.7 km; Fig. 2e), and positively correlated when between 40 and 200 km from roads (mean = 128.8 km). Caribou were also predicted to occur within 100 km of the mean spring sea ice extent (Fig. 2f). Distance to community was ranked as the 8th-most important predictor (Table 1) and the model predicted caribou to occur beyond ~ 20 km from the nearest community (Additional file 1: Figure S1). Mean distance to community was 77.7 km for predicted presences as compared to 58.9 km for predicted absences.

Spatial projections predicted the majority of animals during the calving season to be concentrated in ~ 25,000 km^2^ of land surrounding the upper Colville River (Fig. 4b). Areas of high RIO trailed to the south as far as western Kotzebue Sound but in decreasing breadth and intensity. The calving model accuracy was deemed ‘excellent’ (AUC = 0.98; Overall Accuracy_test_ = 93.53%, Mean Residual_2018_ = 0.05 ± 0.06) using a balanced presence/absence threshold of 0.62. During the calving season, the presence of caribou was most-closely associated with PET (Table 1), especially in areas where PET was less than 260 mm (Additional file 1: Figure S2). Caribou were predicted to occur at distances of 470–600 km from the mean September sea ice extent (Additional file 1: Figure S2). Distance to roads was also influential in the calving model (Table 1) with caribou tending to occur in areas 100–250 km from roads (Additional file 1: Figure S2). Distance to communities was ranked 7th in relative importance (Table 1) and the model predicted caribou to be negatively associated with communities at distances less than 80 km (Additional file 1).

During the insect relief season, models predicted nearly all caribou to occur north of the Noatak River, with most concentrated in an area near the coast but east of Point Hope (Fig. 4c). Two ‘tails’ extended on either side of the Colville River; 1 running along the north side of the DeLong Mountains as far east as Gates of the Arctic National Park and Preserve (GAAR), and the other farther onto the North Slope. Model accuracy for the insect-season model was ‘excellent’ (AUC = 0.98; Overall Accuracy_test_ = 93.84%, Mean Residual_2018_ = 0.09 ± 0.06) at a balanced threshold of 0.75. The most important predictor of high RIO values in the model was PET (Table 1), which was directly correlated with caribou in areas with an average evaporative potential of < 225 mm (Additional file 1: Figure S3). Precipitation was also important (Table 1) and directly related to high RIO in areas receiving an average of > 85 mm in fall (Additional file 1: Figure S3). There was also a disassociation between human infrastructure and caribou during the insect season, where caribou were not generally predicted to occur within 20 km of roads or within 53 km of communities (Fig. 2e, Additional file 1: Figure S3). Distance to the coast also influenced the model (Table 1) and indicated a roughly inverse linear relationship between caribou and the coast, with caribou predicted to occur in areas more than 70 km from the Bering Sea (Additional file 1: Figure S3).

During the late-summer season, the model predicted caribou over a broad area extending from the DeLong Mountains to north of the Colville River (Fig. 4d). Longitudinally, the area reached from east of Point Hope nearly to the community of Anaktuvuk Pass in the central Brooks Range. Other areas predicted as presences included much of NOAT and the northwestern portion of GAAR. The best model for the late summer season (AUC = 0.97; Overall Accuracy_test_ = 87.02%, Mean Residual_2018_ = 0.11 ± .06) had ‘good’ accuracy using a balanced threshold of 0.75. The most important environmental variable in the late-summer model was fall snow-day fraction (Table 1) with caribou tending to occur in areas where snow occurred on the ground more than 70% of days in fall (Additional file 1: Figure S4). Winter precipitation was also influential in predicting caribou and was positively related to presences in areas averaging more than 55 mm in winter (Additional file 1: Figure S4). PET was somewhat less predictive in this model but was correlated with caribou presence in areas where average annual PET was 200 mm – 300 mm (Additional file 1: Figure S4). Caribou were also predicted to occur in areas at least 40 km from roads (Additional file 1: Figure S4). Winter snow day fraction was somewhat important (Table 1) and corresponded with the presence of caribou where snow was typically on the ground more than 90% of winter (Additional file 1: Figure S4). Distance to communities ranked 7th in relative importance (Table 1) and was positively correlated with predicted caribou occurrence from 12 to 144 km away (Additional file 1: Figure S4).

The spatial distribution of the fall model shows wide geographic variation across predicted presence areas (Fig. 4e). The core distribution during this period was predicted to occur from near the community of Kobuk in the north, to the south and west around the margins of Kotzebue Sound, to Bering Land Bridge National Preserve (BELA). Other areas with high RIO included a swath along the western boundary of Kobuk Valley National Park (KOVA) to Kiana and to the east of Kotzebue Sound, a second band ran along the coast from Wainwright to Kotzebue, while other predicted presence areas were scattered across the eastern half of NOAT and the North Slope (Fig. 3e). The pooled fall model predicted the presence of caribou with ‘good’ accuracy (AUC = 0.91; Overall Accuracy_test_ = 83.44%; Mean Residual_2018_ = 0.30 ± 0.13) using a balanced threshold of 0.47. A combination of numerous predictors contributed to the model, but several climatic factors were most important (Table 1). The top variable, PET, was predictive of caribou in areas where average annual evaporative potential was 225–365 mm (Fig. 3a). The mean evaporative potential in presence areas was 294.5 mm versus 368.9 mm in absence areas. The occurrence of caribou was also strongly associated in areas where mean annual ground temperatures were between − 2 °C and − 5.5 °C (Fig. 3b). Growing season length was also highly predictive of caribou in fall in areas with between 130 and 150 days (Fig. 3c). Fall and winter precipitation were both influential in the model, with caribou predicted to occur in areas with between 90 and 145 mm of precipitation in fall and between 44 and 125 in winter (Fig. 3d-e). Roads were ranked 6th (Table 1) and at distances less than 180 km away, there was no clear association with predicted caribou presence and roads in fall (Fig. 3f). The mean distance to roads in predicted presence areas was 116.6 km as compared to 88.0 km in absence areas. Distance to communities was somewhat positively associated with caribou at distances less than 75 km (Table 1, Additional file 1: Figure S5) and the mean distance from communities was 63.2 km for predicted presences versus 60.4 km for predicted absences. The geographic niche of WAH caribou in fall was also weakly associated with minimum September sea ice 450–660 km away (Additional file 1: Figure S5).

In winter, models indicated that the highest predicted RIO were concentrated in BELA and eastward across the Seward Peninsula (Fig. 4f). However, sporadic clusters of predicted presences also occurred throughout GAAR, NOAT, Cape Krusenstern National Monument (CAKR) and across the western North Slope. The winter model predicted presences with ‘excellent’ accuracy (AUC = 0.97; Overall Accuracy_test_ = 92.40%; Mean Residual_2018_ = 0.12 ± 0.09) using a balanced threshold of 0.65. Winter snow-day fraction was the dominant environmental predictor in the model (Table 1) and was negatively associated with the presence of caribou in areas where snow was typically on the ground more than 99% of days in winter (Additional file 1: Figure S6). Other predictors including distance to roads, distance to mean minimum sea ice, PET also contributed to the model’s prediction of caribou (Table 1). Predicted RIO was higher when caribou were between 130 and 215 km from the coast (Additional file 1: Figure S6), whereas caribou were not generally predicted to occur within 38 km of roads (Additional file 1: Figure S6). Distance to community was ranked 6th in importance (Table 1) by the model which predicted caribou to occur at distances between 45 and 108 km from communities, but the 2 were negatively associated outside of this range (Additional file 1: Figure S6).

### Annual migratory models

**Fig. 5:**
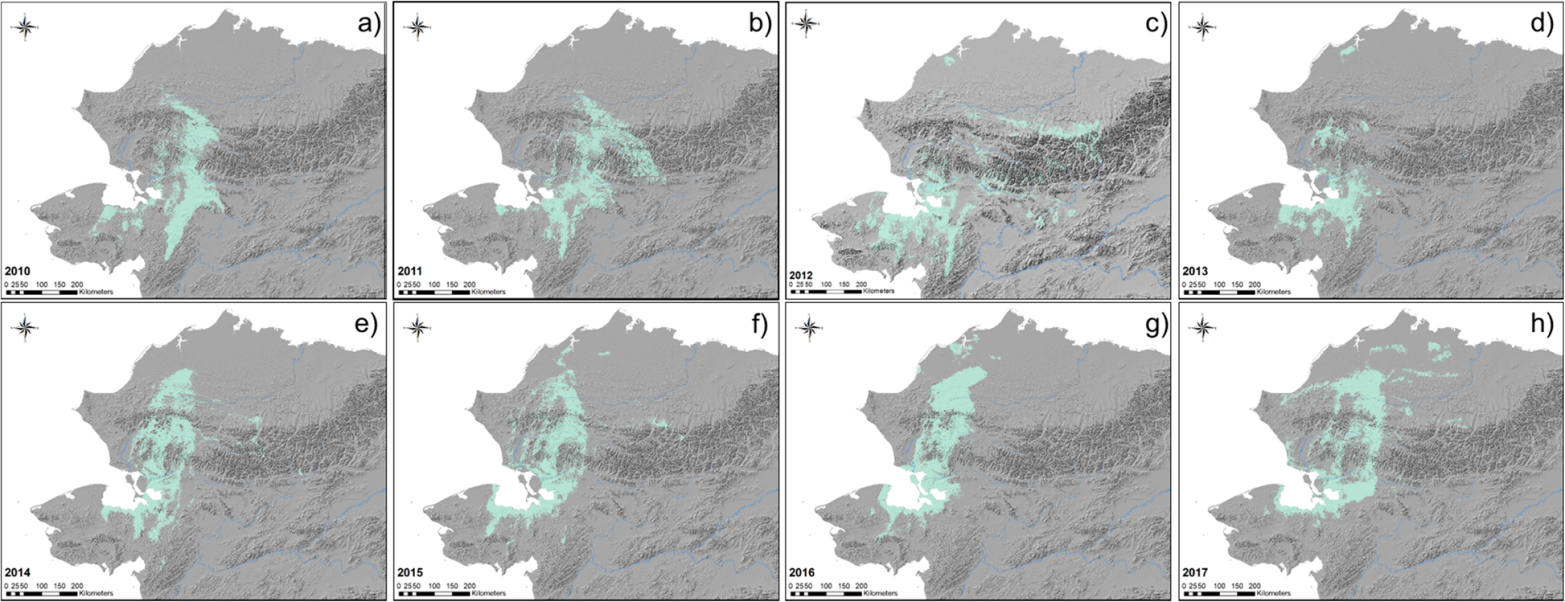
Modeled distributions of Western Arctic Herd caribou during the spring migration season. Models were developed for each year from 2010 to 2017 (**a**–**h**). Shaded area denotes areas of predicted presence based on respective balanced presence/absence thresholds that maximized both sensitivity and specificity

**Fig. 6:**
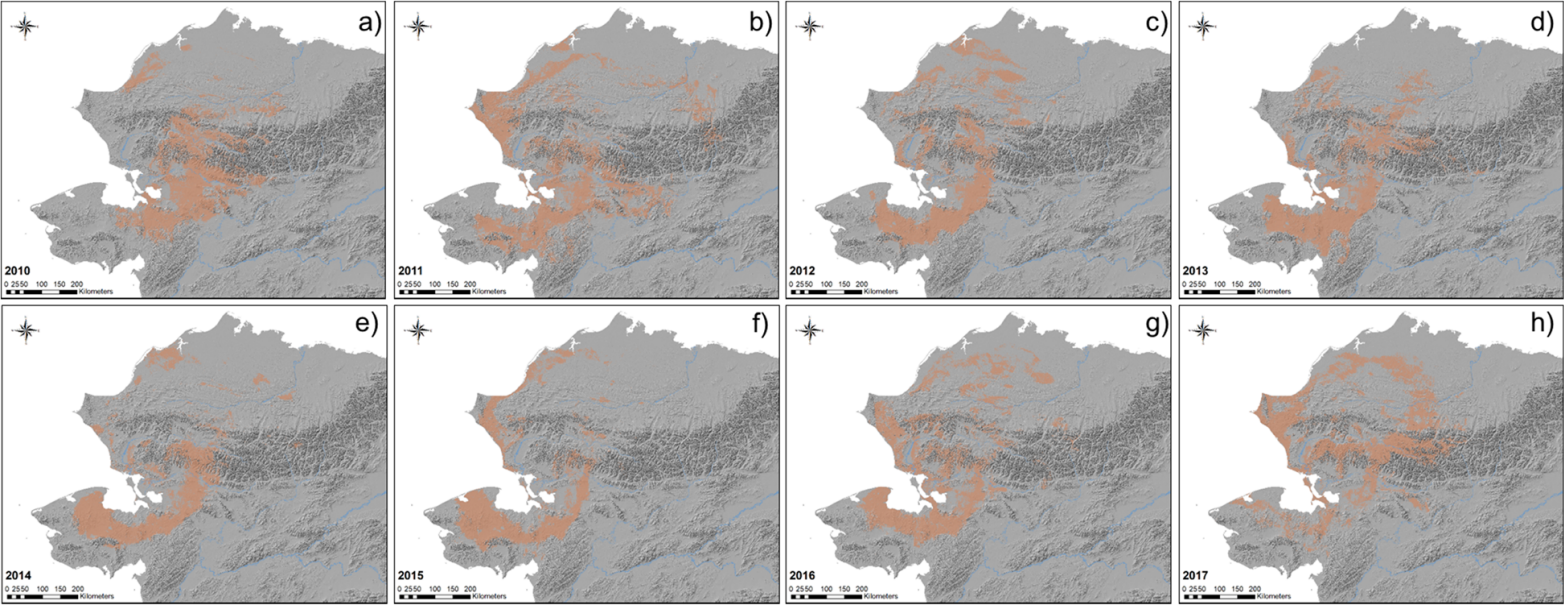
Modeled distribution of Western Arctic Herd caribou during the fall migration season. Models were developed for each year from 2010 to 2017 (**a**–**f**). Shaded area denotes areas of predicted presence based on respective balanced presence/absence thresholds that maximized both sensitivity and specificity

**Fig. 7:**
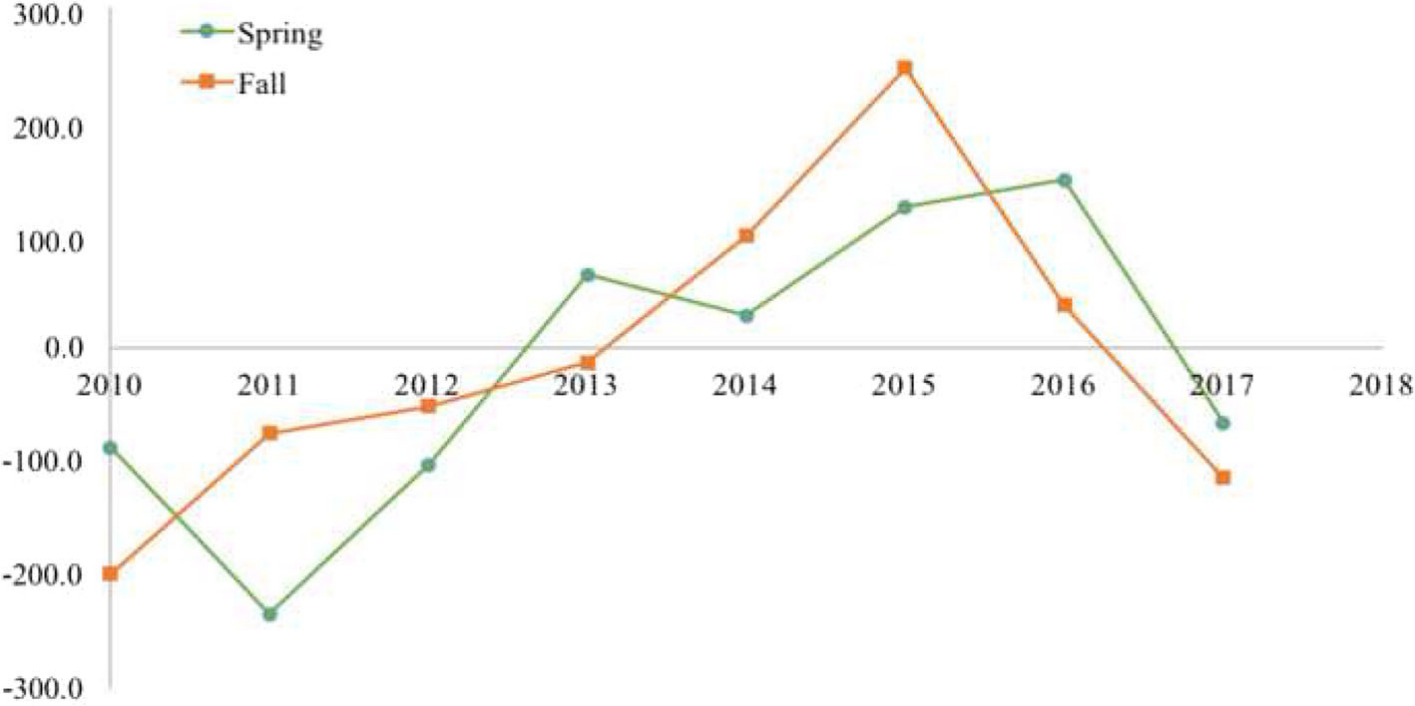
Annual migratory deviation of the Western Arctic Herd from pooled modeled longitude. Chart shows the mean deviations (km) for areas predicted as presences for the spring and fall distribution models of caribou. Negative distances indicate deviation to the east, whereas positive distances indicate deviation to the west of the mean longitude for the pooled fall and spring models, respectively

Annual models during migration seasons provided the means to identify areas of repeated use representing favored migratory habitat. The size of predicted distributions varied annually in size between 21,641 km^2^ and 74,071 km^2^ between 2009 and 2017 (Table 2). Annual predictions of migration patterns varied geographically over the study period with the predicted core of the WAH shifting longitudinally from year to year. We documented an easterly trend in predicted distribution prior to 2012 that became westerly in subsequent years (Figs. 5, 6, 7). Spring models ranged from between 235 km to the east and 148 km to the west of the mean longitude and between 199 km to the east and 246 km to the west of the fall model mean (Table 2, Fig. 7). The average latitude varied somewhat between 97 km south and 58 km north of the pooled spring model mean and between 75 km south and 112 km north of the pooled fall model mean (Table 2).

**Table 2:** Geographic areas and observed latitude and longitude for annual and pooled (2009–2017) distribution models for Western Arctic Herd caribou

Model	Predicted Area (km^2^)	Mean Observed Longitude (°W)	SD	Deviation from Mean (km)	Mean Observed Latitude (°N)	SD	Deviation from Mean (km)
Spring 2010	34,327	159.39317	1.9	88.0 E	66.38077	1.0	75.0 S
Spring 2011	37,240	158.07553	2.3	234.9 E	66.67267	1.0	42.4 S
Spring 2012	42,357	159.25842	4.2	103.0 E	66.41471	1.2	71.2 S
Spring 2013	21,641	160.76047	1.5	64.5 W	66.38118	0.9	74.9 S
Spring 2014	38,725	160.43486	2.2	28.1 W	67.19233	1.2	15.5 N
Spring 2015	38,914	161.29370	2.2	123.9 W	66.93916	1.2	12.7 S
Spring 2016	41,583	161.50840	1.8	147.8 W	67.55609	1.5	56.1 N
Spring 2017	47,198	159.58935	2.7	66.1 E	67.74343	1.4	77.0 N
Spring (2010–2017)	61,095	160.18242	2.6	–	67.05322	1.3	–
Fall 2010	55,941	158.39526	2.3	199.3 E	67.04180	1.1	13.0 N
Fall 2011	74,071	160.00940	3.9	75.4 E	67.13202	1.4	23.1 N
Fall 2012	56,131	160.22082	2.4	51.8 E	66.98286	1.6	6.4 N
Fall 2013	56,684	160.56695	2.8	13.2 E	66.59784	1.2	36.5 S
Fall 2014	49,026	161.57044	3.1	98.7 W	66.57247	1.2	39.3 S
Fall 2015	45,176	162.89438	2.6	246.3 W	66.48665	1.3	48.9 S
Fall 2016	61,165	161.02317	3.0	37.6 W	67.12672	1.4	22.5 N
Fall 2017	66,510	159.65528	3.7	114.9 E	67.92607	1.1	111.6 N
Fall (2010–2017)	118,415	160.68562	3.2	–	66.92524	1.4	–

Deviations from the mean (and standard deviation) were calculated from observed telemetry locations of caribou and are the difference between each annual model’s mean latitude and longitude and the mean latitude and longitude of presences predicted in the pooled model

**Fig. 8:**
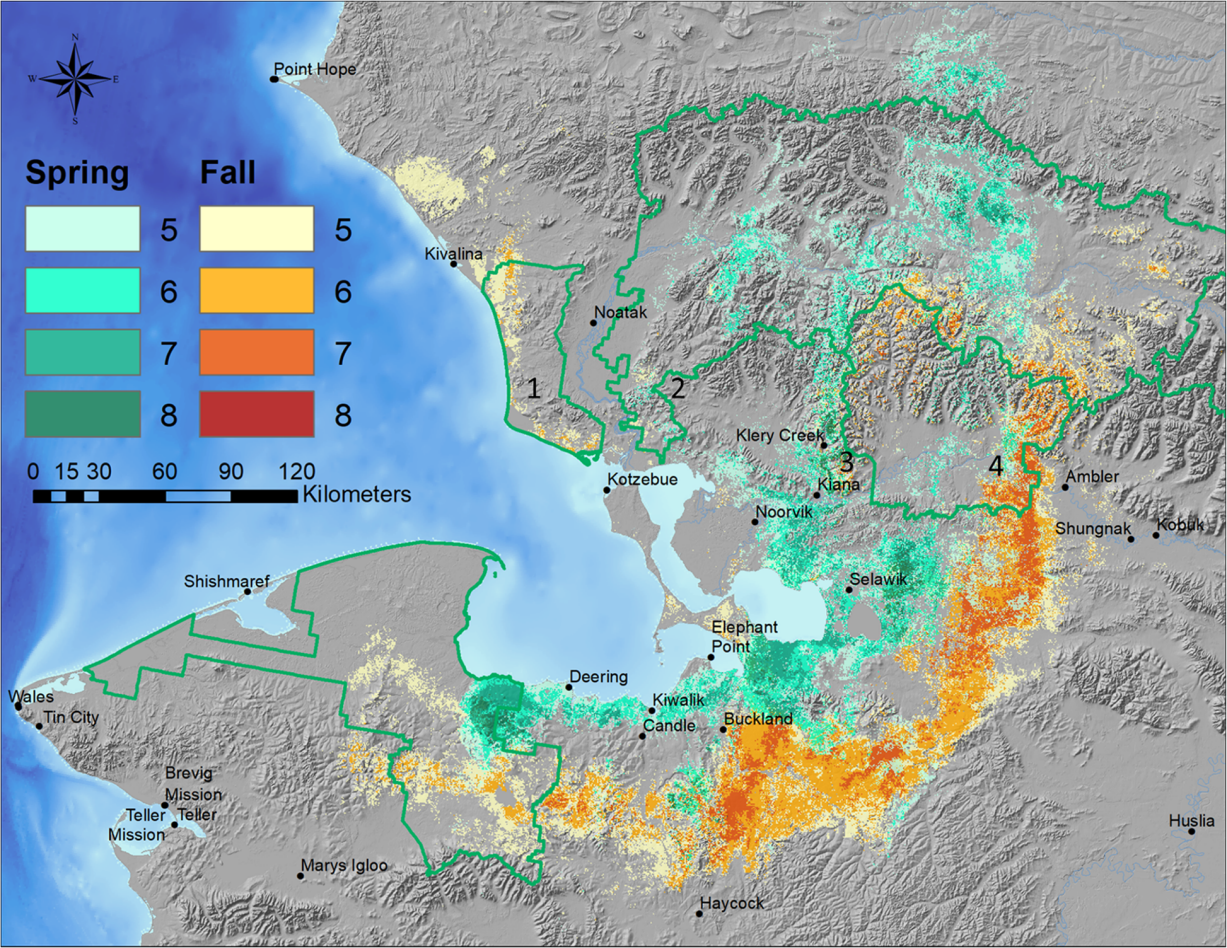
Summed annual distribution models for Western Arctic Herd caribou. Composite models were developed for the spring and fall seasons. Values indicate the total number of years that each pixel was predicted as ‘present.’ Numbers (1–4) indicate main areas of use by caribou during the spring (green) and fall (brown) migratory seasons. National Park Service boundaries are indicated in green

We summed the annual binary model predictions to identify 4 frequently used habitats (Fig. 8). Areas consistently predicted as presences during both seasons in 5–7 of the 8 years included the area along the western extent of the Baird Mountains (Area 2), the uplands along the western boundary of KOVA near Kiana (Area 3), and along the eastern boundary of KOVA west of Ambler (Area 4). Area 1 which traverses the CAKR coastline past Kotzebue was mainly used in fall, and Area 2 which crosses the Kobuk River north of Noorvik was used primarily in spring. Each year at least 1 alternative migratory area was used in addition to the primary one, however south of the Kobuk River, predicted presences in each year converged on a single main area arcing around Kotzebue Sound and west into BELA. Through this area, models predicted caribou farther inland during the fall than in the spring (Figs. 4, 5, 6). Notably, models did not predict caribou to occur in the lower Noatak valley in most years during either fall or spring.

### Land cover use

The specific land cover types selected by caribou in the fall and spring models varied slightly from year to year depending on the primary migration geography (Fig. 1, Table 3). However, the ranking of mean spring (2010–2017) selected land cover types closely matched the rankings for the pooled spring model (Table 3). In spring, caribou selected (> 1.00) for tussock tundra, tall shrub, and herbaceous (mesic) land cover types, in that order. Use of dwarf shrubs was predicted to occur in proportion to its availability (1.00), whereas lichen was used less than expected based on its availability (0.81–0.85). Rankings of land cover selection were the same as for the pooled spring model and the mean of the annual spring models. Similarly, caribou in the pooled fall model selected tussock tundra, tall shrub, and fire scar cover types (Table 3). Mean land cover use in the annual fall models was the same as the order of the pooled fall model, although they differed in that herbaceous cover types were predicted to be used less than expected (0.42–0.48). Low selection indices for urban areas, ice/snow, mixed forest, and spruce forest in both the spring and fall pooled models reflect the lack of predicted use of this land cover type by caribou during migration (Table 3).

**Table 3:** Land cover selection rankings for modeled WAH caribou distributions

Landcover Type	Available Area	Spring Use	Spring Mean Use	Spring Selection	Spring Mean Selection	Fall Use	Fall Mean Use	Fall Selection	Fall Mean Selection
Dwarf Shrub	304,000	34,172	20,801	1.00	1.00	35,719	14,808	0.89	0.89
Herbaceous (Mesic)	276,234	32,774	19,888	1.05	1.05	17,377	6460	0.48	0.42
Tussock Tundra	126,671	25,711	14,883	1.80	1.69	48,779	20,615	2.92	2.93
Spruce Forest	87,892	2358	1863	0.24	0.32	5403	2582	0.47	0.51
Tall Shrub	21,372	2940	2052	1.22	1.46	5173	2452	1.84	2.12
Freshwater or Saltwater	19,411	1018	617	0.46	0.45	1228	701	0.48	0.68
Mixed Forest	18,670	132	127	0.06	0.10	332	203	0.14	0.18
Lichen	11,674	1072	681	0.81	0.85	1284	520	0.84	0.86
Deciduous Forest	11,452	257	197	0.20	0.27	337	211	0.22	0.34
Sparse Vegetation	9462	556	278	0.52	0.42	698	354	0.56	0.68
Fire Scar	8354	380	296	0.40	0.55	1716	730	1.56	1.38
Bareground	3417	106	72	0.27	0.29	315	92	0.70	0.48
Ice-Snow	433	5	3	0.11	0.11	10	3	0.18	0.12
Urban, Agriculture, Road	62	0	0	0.07	0.01	2	0	0.19	0.08

Habitat selections are percent of each land cover type [58] predicted to be used by caribou, divided by the percent area of each land cover type available in the study area. Selected values greater than 1.0 indicate selection for a particular land cover type. Data are for the pooled spring and fall models, and means of individual annual spring and annual fall models during 2010–2017. Available and predicted use areas are in km^2^

## Discussion

We developed ecological niche models that accurately predicted the relative occurrence of WAH caribou over 8 years based on relationships with 37 environmental predictors. We used these models to make inference to the geographic fidelity of caribou migrations, to identify non-linear thresholds in the environmental conditions most associated with predicted caribou distributions, and to rank their use of specific land cover types during migration. TreeNet’s recursive decision tree analysis allowed us to parse and predict this complex and interactive system. Important predictors in most models were primarily associated with 1) climate, 2) coastal features, and 3) human infrastructure. Our analysis also provides detailed and spatially-precise predictions of the migratory tendencies of WAH caribou and what environmental conditions are associated with movement patterns.

### Migratory areas

For the first time, we were able to quantitatively predict the repeated use of the major WAH migratory areas during spring and fall at a 500 m scale. The 4 main areas used during spring and fall were along both the western and eastern boundaries of KOVA, the western edge of the Baird Mountains, north of Noorvik, and along the west coast through CAKR. These areas were used with varied intensity and frequency, but all were used at least 5 of the 8 years of the study period. Seasonal distribution models developed here provide the highest resolution predictions of spatio-temporal patterns and variation of the WAH, while remaining consistent with other prior distribution estimates (see [33]).

Cross-validated model accuracy was excellent for the more stationary seasons (calving, insect-relief, and winter), and these models were also spatially accurate when evaluated against the 2017–18 seasonal WAH telemetry dataset (Mean Residuals < 0.12). Model accuracy during the more transitory periods (spring, fall, and late summer) was good, but models for spring and fall did not predict as well to the 2017–18 data. Their good internal model accuracy (88.45 and 83.22% respectively), yet high mean residuals and large standard deviations (0.39 *± 0.30* and 0.30 *± .13*, respectively), are further evidence of the large inter-annual variability in migratory distributions of the WAH.

In a similar investigation, Nicholson et al. [7] used a Brownian-Bridge analysis to determine that caribou in the CAH also used a variety of routes each year, reflecting variability in individual navigation and decision making. They found, similar to our models for the WAH, that despite annual variability in distributions, specific areas were used repeatedly by caribou. These results, taken with the longitudinal and latitudinal variation from year to year, support the notion that caribou are adaptable in the location and timing of migration, and responsive to local conditions [59], while retaining a collective memory of landscapes over time [60, 61]. This also implies that while certain areas may be favored by caribou over the long-term and used repeatedly, migration and ultimately harvest success in any given year will vary substantially. For example, in 2010 when the mean distribution of the WAH in fall was farthest to the east, communities around Kotzebue Sound (e.g., Noatak, Kiana, and Selawik) were unable to harvest any caribou that year [34]. Since then, the mean longitude of caribou during migration has trended to the westward extreme before returning to the mean during the final year of our study. Successful management of the WAH should recognize this full range of geographic potential, while focusing conservation efforts on the most-frequently used migratory areas outlined above.

### Climate

Based on the variable importance rankings and partial dependence plots for individual predictors (Table 1, Fig. 2, Additional file 1), WAH distribution during spring migration was largely predicted by 4 climatic variables (winter precipitation, PET, fall temperature, and snow-free date) all of which help to determine snow conditions across the region during migration. Winter precipitation and PET were important predictors in both spring and fall, and migrating caribou were predicted to occur in areas with low to moderate levels of winter precipitation and moderate PET. The importance of potential evapotranspiration in all of the seasonal models highlights a strong association between the WAH and broad-scale abiotic factors that determine PET across the region, including temperature, humidity, solar radiation, wind, and biomass. This association corresponds with other results that demonstrated increasing herd size during positive (warm) phases of the PDO and a decreasing population during periods of low (cool) PDO [45]. Areas in Alaska with the lowest PET occur primarily north of the Brooks Range and on the Seward Peninsula, compared to the warmer, drier conditions more common to interior Alaska. Likewise, most of the WAH range coincides with an area of northwest Alaska that receives moderate winter precipitation (50–120 mm) as compared to the higher totals found at the highest elevations or in the ranges of other arctic caribou herds. Areas receiving moderate amounts of fall precipitation or cool mean annual ground temperatures were also associated with caribou distribution in fall, corresponding to a similar area encompassing the northern Seward Peninsula, western Brooks Range, and western coast where caribou occur during fall. At the local scale, when posed with a range of available conditions in the Brooks Range, caribou were predicted to occur in valleys or west-facing ridges, where PET was higher and snow cover was shallower than higher elevations and on east facing aspects.

Earlier snow free dates were also highly predictive of caribou in spring, whereas a longer growing season was predictive of caribou in fall. In contrast to PET and winter precipitation trends which vary primarily across wider scales, the tight correspondence between early snow free date and areas predicted to have been repeatedly used by caribou in spring is apparent at a much finer local scale (Additional file 1) [62]. Snow cover has previously been shown to affect the timing and habitat selection of migrating caribou as they seek out patches of emergent forage where snow has melted [23, 62, 63]. Caribou have evolved exceptionally efficient locomotion such that the energy required to move is exceeded by the amount of forage they are able to obtain from new patches of high quality forage [64]. However, sinking through deep or crusted snow increases energy expenditure exponentially, so encountering such conditions during migration could cause delays that result in phenological and geographic mismatches during calving that could ultimately lead to starvation. This may explain why caribou were not predicted to occur in areas of late snow melt (Fig. 2d) and were predicted to travel closer to the coast in spring (Fig. 8) on wind-packed snow or sea ice. To avoid poor snow conditions, spring migration is often constricted into areas where animals develop packed trails that tend to follow windswept terrain that hardens snow [65, 66] and aids locomotion or other areas with low snow accumulation [64].

Caribou also selected areas of tussock tundra and areas with tall shrubs during migration (Table 3) but did not select forested habitats. Tall vegetation can impede movements, reduce visibility and increase snow depth, which may account for caribou selecting tussock tundra during spring migration. This explanation runs counterintuitive to the predicted selection for tall shrubs, however. Tall shrubs tend to occur in riparian valleys, where caribou were often predicted to occur, so selection for this tall cover type could be coincident with other suitable environmental conditions in Brooks Range valleys. The winter model also showed caribou using areas with less than complete or shallow snow cover (Fig. 3f). Caribou may be using areas which tend to be snow free longer in fall and spring, and which have lower relative snow depths in winter, providing them with better traveling conditions and more accessible forage. Our models predicted WAH caribou to occur in areas where moderate precipitation and relatively warmer temperatures lead to earlier snow melt, and we surmise that these combine to minimize thermodynamic and nutritional stress throughout the year.

### Coastal influences

We also documented a correlation between the predicted occurrence of caribou and coastal features, as evidenced by the moderate importance of the distance to coast and mean sea ice extent predictors in many of the caribou distribution models. In both the fall and spring models, we predicted caribou to occur within 160 km of the coast. In spring, this predicted area of occurrence was directly adjacent to the coast (< 25 km) and spring ice on Kotzebue Sound, where snow is windswept and packed. In fall they were predicted to occur more than 35 km from the coast. This more inland migration would bring them through the warmer, drier areas east of Kotzebue Sound where the growing season is longer. Such a seasonal, clockwise movement may help the herd avoid recently-foraged areas while also extending forage access later into the fall. During the insect avoidance season, caribou were predicted to occur within 60 km of the coast, potentially to take advantage of ocean winds and cooler temperatures that keep insects at bay [67].

The predicted distribution of caribou in spring and fall also corresponded with the proximity to the mean maximum and minimum sea ice extents (Table 1, Fig. 2f Additional file 1). While this relationship could coincidentally stem from the existing geographic range of the WAH near two oceans, the associations between caribou and sea ice extent could also be indicative of the direct or indirect influences of sea ice on climate, land cover, and by extension, on the distribution of the WAH [68]. Sea ice loss has been shown to indirectly affect terrestrial ecosystems through changes in climate that lengthen the growing season, promote the increase of summer plant biomass (as indicated by NDVI) [69, 70] and increase the amount of fall snowfall, which in turn insulates and raises ground temperatures [69, 71]. Associations with the minimum ice extent in both spring and fall were strongest when caribou were between 450 and 750 km away from the ice edge, likely reflecting only the geographic position the WAH in the region. In contrast, the correlation with mean maximum sea ice in spring were evident at much closer distances, indicating the possibility of direct effects as well. Furthermore, telemetry data show that some WAH caribou even traveled over sea ice to cross Kotzebue Sound [72]. The decrease in sea ice extent in arctic Canada has caused Peary caribou to experience an increase in landscape resistance of 15% [73], so the loss of sea ice as a travel medium, much like an increase in snow depth or water content, could theoretically increase the energetic costs of migrating WAH caribou as well [64, 72].

### Human infrastructure

We also showed that caribou were negatively associated with roads at distances less than 20–100 km away during the spring, calving, insect-avoidance, late summer, and winter seasons (although there was no correlation in fall), and distance to roads was among the top predictors in each of these models (Table 1). These trends may reflect the tendency of the WAH to occur in the geographic space between the Delong Mountain Transportation System (also known as the Red Dog Mine road), the road north of the community of Kobuk, and roads emanating from Nome. However, during the insect relief season, some WAH caribou were predicted to use habitat near the coast, placing them in an area closer to the Red Dog Mine road, yet they were still not predicted to occur within 20 km of the road (Fig. 2e). This distance towards the upper end of previously reported zones of influence by roads that extended 14–23 km [74, 75]. Wilson et al. [16] found that 30% of WAH caribou traveling within 15 km of the Red Dog Mine road were delayed by an average of 33 days during fall migration. Moreover, Leblond et al. [76] documented a road-displacement distance of 5 km and that a majority of their study population failed to cross a highway which lead to a loss of > 50% of their range. Our models are consistent with this and other research that show caribou tending not to occur near human infrastructure, including roads, communities, and developed areas [18, 77–79]. For example, the density of females and calves in the CAH was shown to be low within 4–6 km of linear features within the herd’s calving area, although the herd did not avoid the area during other seasons [78, 80, 81].

Our models indicated a dissociation between caribou and towns during the spring, calving, insect-relief, and winter (but not fall) seasons by 20–75 km (Additional file 1), with distance to community ranked among the top 10 predictors of 37 in all models (Table 1). The predicted tendency for caribou to occur away from communities may be best exemplified by their absence around the community of Noatak, but is also visible in the immediate vicinities of Kobuk, Kiana, Noorvik, Selawik, and Kotzebue in models and telemetry records (Fig. 8). Their predicted lack of occurrence near Noatak could also be related to spruce forests found in the valley, as caribou were predicted in treeless land cover types in much greater proportion relative to their availability (Table 2). However, the potential exists for caribou to be disturbed by noise from motorized equipment and airstrips that can be detected as far as 30 km of communities [37, 40], and begs further investigation. In contrast, caribou were weakly associated with communities within 75 km distance during fall, suggesting either that caribou are either less concerned with disturbances during this season, or that following preferred habitat during the southward migration brings them closer to more communities. In other words, communities may have historically been intentionally placed within preferred fall migratory areas, but shifting migratory patterns may be altering this association. While the passage of caribou may still continue in the vicinity of anthropogenic disturbances, the timing and duration of migration can be delayed in developed areas, and broader effects on population health have not yet been demonstrated [16, 26].

### Land cover use

Based on our environmental niche models, we found that caribou were predicted to select lowland tussock tundra, tall shrubs and herbaceous cover types in spring and fall. The selection of tussock tundra and herbaceous cover types are similar to other habitat selection research in the region, however the selection for tall shrubs runs counter to other results [27, 45]. In an analysis of caribou movement through NOAT using step-selection functions, caribou exhibited random-walk behavior, and exploited areas of tussock tundra and dwarf shrubs, while avoiding tall shrubs, rugged terrain, and rivers [27]. Such selection is consistent with caribou maximizing energetic efficiency and seeking out areas of optimal forage, namely patches of exposed lichen in warm microclimates, especially on the south side of the Brooks Range [26, 82, 83]. In fall and winter, caribou obtained ~ 71% of their forage from lichens, whereas the next most important forage types, moss and shrubs comprised just 20% [82]. In fact, lichens typically comprised 65–70% of the diet of migratory caribou with substantial predation pressure in Alaska [31, 82–84]. The accessibility of forage to caribou, especially as related to snow cover and fire, are factors driving movements of the WAH [28, 85].

Our results did not show any major influence of topographic variables (e.g., elevation, aspect, slope, terrain ruggedness) in models for any season or year. However, there was a positive relationship between occurrence and elevation above 200 m and an inverse relationship with terrain ruggedness. [27]. Similarly, caribou in the Porcupine Herd were shown to use corridors at higher elevations but with relatively gentle terrain, while avoiding spruce forests, steep mountains, and wet lowlands [79]. Together these results suggest that caribou follow energetically efficient courses through snow-free areas to minimize locomotive resistance and maximize available forage, while largely conforming to learned directional orientation to guide migration [26, 60].

## Conclusion

We posit that given the influence of climate variables in our models, that rapid changes in these processes will play outsized roles in determining the trajectory of caribou in the future. Increased climatic volatility and annual shifts in migratory patterns of the WAH underscore the need to allow caribou to select among a variety of habitats in order to find the most suitable conditions in a given year or season. Strong links between caribou distribution and interacting trends in snow, PET, growing season, and sea ice are apparent in our models. As the volatility in these and other climate effects (e.g., increased fire frequency and shrubification) grows, WAH caribou migration patterns are also likely to become more variable in the future. We surmise that this could result in changing phenologies (e.g., delayed migration, increased spatial variability in calving) as a means of coping with decreasing climatic reliability. As such, the success of harvesting caribou near any given rural community in a given year may correspondingly decrease in the future as well [86].

It remains unclear whether WAH caribou are capable of adapting to rapid climatic changes or whether future predictions of a distribution contraction will occur. Some potential changes, like longer growing seasons, may aid caribou by allowing for increased energetic intake, while others, like the loss of sea ice from Kotzebue Sound and deeper, crusted snow, could make travel costlier near the coast. As a species, caribou have thus far largely adapted to an evolving suite of highly variable and changing ecological conditions, including new predators [87], changing habitat and forage quality [31], and the development of portions of their range [16]. It is possible that they may also be capable of adapting behaviorally to the threats of accelerating climate change and further limited development. However, the rates of environmental change in the Arctic are unprecedented and the ability of WAH caribou to adapt at these rapid rates should not be taken for granted.

Although we did not explicitly measure displacement, our models predicted the majority of WAH to occur in areas at least 20–100 km from roads and communities during all seasons except fall (Fig. 2, Additional file 1). This could have important implications for the herd should any of the numerous proposed roads be constructed in northwest Alaska, especially those that would bisect the documented migratory areas in the Kobuk Valley (Fig. 8). Proposed roads extending from the communities of Ambler to Kotzebue, Noatak or the Red Dog Mine could have major consequences for caribou because these corridors would bisect all of the main migratory regions. While a proposed road to access the Ambler mining district stops short of much of the core WAH migratory area, it would cut directly through a core area used by caribou during the winter (Fig. 3f). In addition to potential disruptions to migration [16, 21] and the effects of windborne dust [88, 89] resulting from industrial traffic, an associated increase in hunter access to migratory areas could have far more detrimental effects on caribou populations, should this or other new roads be publically accessible (see [26, 90]). Given the extensive climatic and anthropogenic disturbances threatening the Western Arctic Herd, we echo the call for comprehensive management planning that recognizes and conserves a variety of migratory areas to buffer caribou from environmental volatility as part of a long-term persistence strategy for caribou in the Arctic [7, 79].

## Additional file

Additional file 1: Partial dependence plots for the top 30 variables in each of the seasonal predictive models for caribou in the Western Arctic Herd. These are model-based simulations that chart the non-linear relationship of the response over the range of each predictor variable while controlling for the other predictors. (DOCX 533 kb)

## Data Availability

The datasets used and/or analyzed during the study are archived at: https://irma.nps.gov/DataStore/Reference/Profile/2260262.

## Abbreviations

AUC: Area Under the Curve
BELA: Bering Land Bridge National Preserve
CAH: Central Arctic Herd
CAKR: Cape Krusenstern National Monument
GAAR: Gates of the Arctic National Park and Preserve
GPS: Global Positioning System
KOVA: Kobuk Valley National Park
NDVI: Normalized Difference Vegetation Index
NOAT: Noatak National Preserve
PDO: Pacific Decadal Oscillation
PET: Potential Evapo-transpiration
RIO: Relative Index of Occurrence
WAH: Western Arctic Herd
