# Supplementary material for: Using seasonal landscape models to predict space use and migratory patterns of an arctic ungulate
Source: Mov Ecol. 2019 Jun 6;7:18. doi: 10.1186/s40462-019-0162-8 (PMC6551874; doi:10.1186/s40462-019-0162-8)
Supplement: Supplementary file 1 — Partial dependence plots for the top 30 variables in each of the seasonal predictive models for caribou in the Western Arctic Herd. These are model-based simulations that chart the non-linear relationship of the response over the range of each predictor variable while controlling for the other predictors. (DOCX 533 kb) [file 40462_2019_162_MOESM1_ESM.docx]

Additional file A. Partial dependence plots for the top 30 variables in each of the seasonal predictive models for caribou in the Western Arctic Herd.

Figure 1. Partial dependence plots for the top 30 variables in the pooled spring distribution model for caribou in the Western Arctic Herd, in order of importance.

Figure 2. Partial dependence plots for the top 30 variables in the pooled calving model for caribou in the Western Arctic Herd, in order of importance.

Figure 3. Partial dependence plots for the top 30 variables in the pooled insect-relief model for caribou in the Western Arctic Herd, in order of importance.

Figure 4. Partial dependence plots for the top 30 variables in the pooled late summer distribution model for caribou in the Western Arctic Herd, in order of importance.

Figure 5. Partial dependence plots for the top 30 variables in the pooled fall distribution model for caribou in the Western Arctic Herd, in order of importance.

Figure 6. Partial dependence plots for the top 30 variables in the pooled winter distribution model for caribou in the Western Arctic Herd, in order of importance.
